# Experimental study of a cryogenic pneumatic thrower for launching soft projectiles in avalanche protection

**DOI:** 10.1038/s41598-025-26523-x

**Published:** 2025-11-27

**Authors:** A. Tasmukhanova, D. Yerezhep, B. Krutskikh, Y. Korshikov, T. Yerlanov, A. Lesbayev, A. Zhakypov, A. Tukmakova, K. Aimaganbetov, A. Akylbayeva, A. Aldiyarov

**Affiliations:** 1 Al-Farabi Kazakh National University, Al-Farabi Avenue, 71, Almaty, 050040 Kazakhstan; 2 Satbayev University, Satbayev str., 22, Almaty, 050013 Kazakhstan; 3 Almaty Management University, 227 Rozybakiyev str, Almaty, Kazakhstan

**Keywords:** Snow avalanche, Pneumatics, Launcher, Ballistics, Avalauncher, Liquid nitrogen, Engineering, Materials science, Physics

## Abstract

A novel cryogenic pneumatic launcher for remote avalanche initiation has been developed and experimentally investigated. The system uses liquid nitrogen as a working medium, with its explosive evaporation triggered by the heat of a thermite reaction. The rapid phase transition of the cryofluid generates high pressure without sharp shock waves and enables smooth acceleration of soft-bodied projectiles without structural damage. This approach eliminates key drawbacks of conventional avalanche control methods, such as artillery and gas-dynamic Avalauncher systems. A laboratory-scale prototype was built (a 4-meter-long steel barrel with an outer diameter of 89 *mm*) equipped with an optical chronograph to measure projectile velocity (~ 1 *kg*). The internal ballistics were studied for thermite charges of 3–6 g and liquid nitrogen volumes of 0–200 *ml*. It was found that with 3 g of thermite, the optimal performance was achieved by adding ~ 100 *ml* of liquid *N₂*, resulting in a projectile speed of ~ 55 *m/s*, maintained up to barrel exit. Increasing the charge to 6 g of thermite and 200 *ml* of liquid *N₂* raised the peak velocity to over 90 *m/s* and extended the acceleration phase. The results confirm the feasibility and advantages of the proposed. Method projectiles accelerate without damage, and the system ensures high mobility, environmental safety (no fragments), and a controlled pressure profile with sufficient delivery range. The combination of gentle acceleration and operational mobility makes the launcher promising for avalanche control and other applications.

**Supplementary Information:**

The online version contains supplementary material available at 10.1038/s41598-025-26523-x.

## Introduction

The spontaneous release of snow avalanches poses a significant threat to human lives and critical infrastructure in mountainous areas, making avalanche safety a key component of mountain hazard management^1–5^. To reduce the risk of avalanches, controlled artificial triggering has become a primary strategy. This technique originated from military methods used to destabilize snowpacks on steep slopes and has evolved into an essential component of modern civil protection systems^6,7^.

In artificial triggering, different types of solid explosives and explosive gas mixtures are used to initiate snowpack failure. When these charges detonate, they create a shock wave that travels through the snowpack and disrupts its structural integrity, leading to an avalanche^8–10^.

An essential practical challenge in the use of explosive charges is the accurate and safe delivery to the target location. Common methods of delivery include manual placement, aerial delivery by helicopters, conventional artillery, rocket systems, and pneumatic launchers. Each method has specific operational advantages (e.g., range, speed of delivery, or precision), but also has distinct limitations, such as mobility, weight and size of equipment, environmental hazards from projectiles, or mechanical stress on soft-bodied projectiles. A detailed comparison of these methods can be found in literature (see H. Gubler et al.^8^, S. Simoni et al.^11^, E. R. Lachapelle^12^.). This motivates the search for more portable and low-fragmentation delivery systems that are suitable for soft-bodied projectiles.

At first glance, conventional artillery appears attractive because of its operational mobility and capacity for use across a wide range of weather and light conditions. Nevertheless, significant drawbacks arise from the rigid, metallic design of conventional projectiles. Metal shells may penetrate deeply into the snowpack or remain embedded after impact, creating long-lasting hazards to people and wildlife; fragmentation increases the risk of collateral damage to infrastructure and complicates post-operation clearance^7,13–16^. Additionally, metal projectiles raise environmental and logistical concerns related to unexploded ordnance and debris. For these reasons, interest has grown in non-fragmenting, non-metallic projectiles (e.g., plastics and biodegradable composites) that minimize fragment generation and environmental impact while providing comparable delivery performance for avalanche control applications^6,17^.

The adoption of soft-bodied projectiles introduces new technical challenges. Soft materials are highly susceptible to mechanical stress and can fail under rapid pressure transients and high peak accelerations typical of conventional artillery launches. Fragile and non-metallic casings have a strong strain-rate dependence: their apparent stiffness and strength can increase at very high strain rates, but they can also develop localized failure modes such as cavitation, craze formation, brittle fracture, or shell collapse when subjected to sudden pressure changes like those in artillery launches. Experiments and reviews show that dynamic loading in a range of 10²–10⁴ s^− 1^ leads to different failure mechanisms than quasistatic loading, so uncontrolled pressure spikes and high peak accelerations can easily damage soft-bodied projectiles^18,19^.

Therefore, delivery systems for such payloads must ensure gentle launch conditions: a controlled, smoothly rising pressure along the barrel, constrained peak accelerations and reduced jerk, so that internal stresses and strain-rates remain below the material’s failure limits. Producing a near-uniform acceleration profile minimizes stress concentrations and the risk of deformation or rupture prior to exit, and is thus essential for reliable delivery of non-rigid, fragment-free charges in avalanche mitigation. This conclusion is corroborated by recent experimental and numerical studies on shock-mitigation, soft-body impact dynamics, and acceleration-induced pressure-gradient and cavitation phenomena^20–22^.

In recent years, pneumatic systems known as Avalaunchers (manufactured in France or USA) have been widely adopted for launching non-fragmenting projectiles^23^. These Avalaunchers are powered by compressed gas stored in tanks and can launch payloads up to several kilometers away, enabling remote avalanche triggering in hard-to-reach or dangerous locations. This range allows for remote Avalanche triggering in hard-to-reach or dangerous locations. However, there are significant engineering and operational challenges with Avalaunchers. Their use of high-pressure receivers and complex high-speed valves add weight, volume, and maintenance to the system, limiting their deployability and flexibility in remote and high-altitude terrain. The logistics of transporting, storing, and handling heavy compressed gas cylinders also restrict field operations, especially in inclement weather or difficult access conditions. Furthermore, the rapid release of compressed gas creates short, intense pressure surges and waves within the barrel, which can be difficult to control. These transient phenomena can cause large peak accelerations and stress gradients, which can be harmful to soft, thin-walled, or otherwise soft-bodied projectiles. Similar issues have been explored in studies on the dynamics of compressed gas-powered launchers, where temporary overpressure and wave reflection significantly impact the integrity of the projectile and its internal ballistic stability^24–26^.

Thus, despite the diversity of existing solutions, none fully overcome the inherent trade-offs between mobility, system mass, maintenance complexity, and the generation of high peak pressures and accelerations. Consequently, there remains a clear need for alternative launcher concepts that combine the advantages of established pneumatic and gas-dynamic systems while minimizing their structural, operational, and dynamic limitations.

This paper reports the design, construction and experimental characterization of a laboratory prototype cryogenic pneumatic launcher that uses a cryogenic liquid as the working medium. The proposed concept emphasizes compactness, mobility and operational simplicity compared. Its working principle exploits the energy released during an abrupt liquid-to-vapour phase transition: a fast-reacting thermite charge locally atomizes and superheats the cryogen, triggering rapid evaporation and two-phase expansion that generate the driving pressure for projectile acceleration.

The methods of creating pressure in such systems are determined by the interaction of a hot body and a liquid. When a hot particulate or reactive front contacts a volatile or subcooled liquid, near-wall heat flux, hydrodynamic drag and the prevailing boiling regime (film vs. nucleate) determine whether evaporation proceeds gradually or undergoes a transition to explosive boiling/phase explosion. Classic experiments on boiling over moving hot bodies demonstrate how drag and heat-transfer coefficients co-evolve with the boiling state and flow separation, thereby controlling the rate of pressure rise in the surrounding fluid^27^. Later studies have shown that film-boiling stability in subcooled liquids is strongly pressure-dependent^28^, and that thermal coupling between molten/hot bodies and liquids can lead to hydrodynamic fragmentation and vapor-explosion triggering via interfacial instabilities and heat-transfer spikes^29^. Recent experimental work emphasizes that small changes in superheat, confinement or perturbations may precipitate runaway vapour generation^30^.

In our device the thermite acts as a localized high-temperature source: it fragments and superheats liquid nitrogen (*LN*_*2*_)^31–33^, thereby triggering a rapid pressure rise. The investigation of this process is ongoing and requires further experimental and theoretical study.

The results of an experimental study of the internal ballistics of the developed installation are presented. The primary objective of this work is to experimentally validate the feasibility of cryogenic liquid for gentle acceleration of non-fragmenting projectiles and to characterize its internal ballistic parameters. Mass-dimensional models made of polyethylene terephthalate (PET) were used as throwing objects, which corresponds to the mass-dimensional parameters of practical shells for initiating avalanches^15^. To assess the reproducibility and characteristics of internal ballistics, a series of laboratory tests were conducted with various combinations of the mass of the thermally active component (thermite) and the volume of *LN*_*2*_.

## Experimental installation

**Fig. 1:**
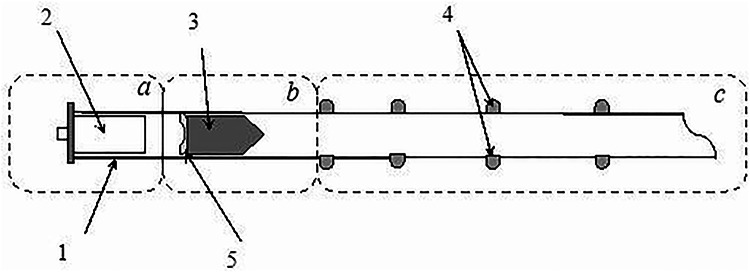
Schematic diagram of a cryopneumatic launcher: **a** – propellant charge assembly; **b** – loading chamber; **c** – measuring Sect. 1 – barrel; 2 – cartridge case; 3 – projectile; 4 – optical chronograph sensors for recording flight time; 5 – obturator.

Figure 1 shows the basic diagram of the installation. The main element of the pneumatic system is the barrel *1*, made in the form of a smooth steel pipe 4 m long. The outer diameter of the pipe is 89 *mm*, and the wall thickness is 3.5 *mm*. For ease of description, the barrel part of the installation is conventionally divided into three parts: the pressure generator *a*, the charging chamber *b* and the working measuring part *c*.

In the pressure generator *a*, the propellant charge is placed in a special sleeve *2*. The part in which the projectile *3* is placed is called the loading chamber *b*. The installation of the projectile in the loading chamber is carried out freely, without special fixation. The projectile is held by the obturator *5*, which, in addition to fixation, also prevents gas pressure losses behind the projectile along the barrel. Since the shell of the projectile is made of a soft material, its installation does not require additional sealing. Thus, the obturator performs the function of the main projectile retainer inside the tube within the loading chamber.

An optical chronograph *4* is installed in the measuring section ***c***, designed to record the moments of the projectile’s flight. The chronograph comprises eight optical sensors utilizing infrared diodes as their active elements^34^. The diodes are arranged in pairs along the housing, 500 mm apart and diametrically opposed to each other. In principle, the diodes can serve as both a source and receiver of the signal, so only one type of diode is used here. Infrared diodes were also chosen for their reduced sensitivity to smoke and vapors generated during the explosive vaporization of the thermite-nitrogen mixture^35^. The type of LEDs used is BL-L513IRAC with a wavelength of 940 nm. It has a typical response time of 0.2–0.3 *µs* and the sensitivity of the receiving part of the system at this length reaches 0.6 *A/W*. In addition, such a system does not affect the movement of the projectile and is not damaged after its flight, which is especially convenient with a large number of recording channels and allows to quickly conduct a series of experiments. The principle of constructing an optical chronograph or, as they are otherwise called, photoelectric recording systems is based on the use of modulation of the light flux falling on the element sensitive to illumination^34,36^.

**Fig. 2:**
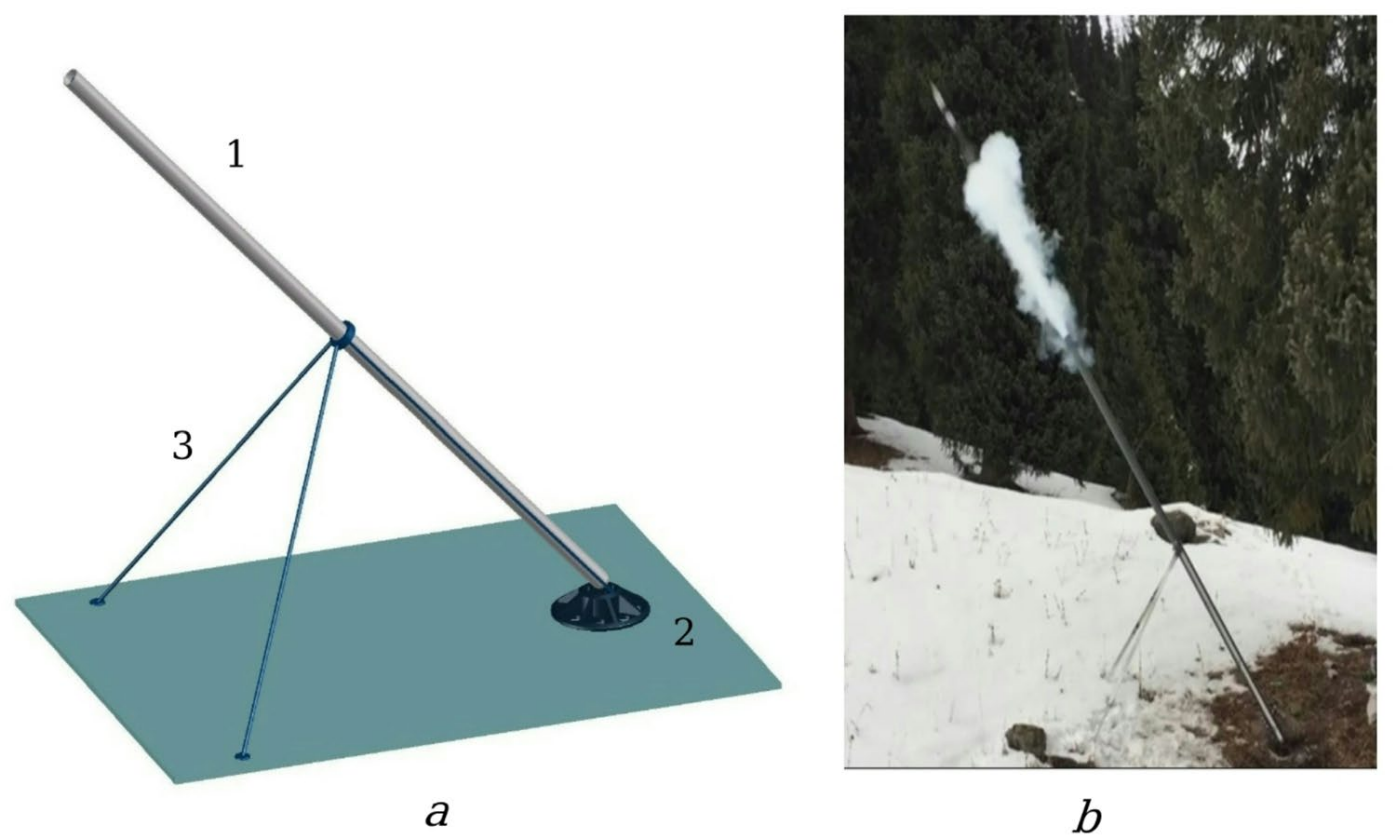
Experimental cryopneumatic installation: **a** – sketch of the design (1 – barrel; 2 – support plate; 3 – two-legged support), **b** – actual frame of the installation in operation at the testing ground.

Thus, the proposed cryopneumatic installation is a special case of a mortar gun, which, as is known, consists of three main parts: a barrel, a support plate and a two-legged support on which the barrel is mounted, as shown in Fig. 2a. The barrel *1* rests on a massive support plate *2*, which takes the entire recoil force and transfers it to the ground, providing stability during firing. The two-legged support *3* holds the barrel in the desired position and allows changing the elevation angle (vertical guidance) and rotation angle (horizontal guidance). Figure 2b shows a frame recorded at the moment of firing a projectile by a cryogenic pneumatic installation in field conditions.

## Operating principle of a cryogenic pneumatic installation

To accelerate the launched body, it is necessary to create high pressure, regardless of whether gas-dynamic or alternative methods, such as electromagnetic ones, are used. Pneumatic throwing devices provide this pressure due to the energy of compressed gas, which acts directly on the projectile and transfers momentum to it. Unlike powder and explosive systems, in this case, chemical combustion and the release of a large amount of heat do not occur. Energy is supplied to the working gas in such systems by means of its preliminary compression using a piston, or from a high-pressure receiver.

As we have already noted above, in the proposed installation for creating high pressure in the barrel, instead of compressed gas, the energy arising from the rapid evaporation of the cryofluid is used (energy calculation is provided below in the section “4. ENERGY BALANCE ASSESSMENT”). The experimental fact of transient processes occurring during explosive evaporation of the cryofluid at the overheating limit leads to the formation of a vapor phase of the cryofluid with a rapidly increasing pressure^32,33^. It is believed that the mechanism of all these explosions is the extremely rapid evaporation of liquid at the limit of overheating as a result of depressurization or contact with a hotter substance. It should be noted that in our case, the initiator of instantaneous evaporation of cryofluid is a standard fast-reacting thermite mixture based on aluminum and iron oxide. We used *LN*_*2*_ with a purity of 99.5*%* as a cryofluid.

### Mechanisms of explosive boiling of LN_2_

The rapid vaporization and accompanying macroscopic effects observed in this study (sharp pressure spikes, acoustic emissions, etc.) are interpreted within the framework of the classical theory of boiling regimes and vapor explosion initiation mechanisms^30,37^. From the standpoint of hydrodynamics and heat transfer, three characteristic regimes are distinguished: nucleate boiling, stable film (Leidenfrost) boiling, and the transitional regime, in which the vapor film locally loses stability and collapses, giving rise to intense nucleation^38,39^. The key mechanism of vapor explosion initiation described in the literature is associated precisely with the abrupt transition from the film to the nucleate regime and the simultaneous sharp increase in the interfacial contact area between the superheated surface and the liquid.

Thus, in the proposed setup, the explosive evaporation of *LN*_*2*_ is initiated by the rapid heat release from the thermite mixture, leading to a vapor explosion characterized by boiling at the superheat limit. This process corresponds to the theory of Boiling Liquid Expanding Vapor Explosion^15,40,41^, in which a superheated liquid, upon contact with a hotter substance, undergoes a rapid phase transition that results in explosive expansion and a sharp pressure rise^38,42^. In our configuration, the thermite acts as a thermal trigger, while *LN*_*2*_ (boiling point ~ − 196 °C at 1 *atm*) reaches the superheat limit (~ 90–100 *K* above saturation), promoting homogeneous nucleation and rapid vapor formation^43–45^.

The boiling regime of *LN₂* proceeds through several stages depending on the heat flux (q) and the temperature difference (*ΔT = T*_*hot*_ *− T*_*sat*_). Initially, upon contact with the hot thermite combustion gases, at high ΔT, film boiling occurs, forming a stable vapor layer that insulates the liquid (the Leidenfrost effect)^28,46^. However, turbulent mixing caused by the thermite combustion disrupts this film, leading to transitional boiling with intermittent liquid–vapor contact and, ultimately, to explosive nucleate boiling once the superheat limit is exceeded. This results in homogeneous nucleation throughout the *LN₂* volume, where vapor nuclei form spontaneously, causing fragmentation into fine droplets and greatly accelerating evaporation^31,38,47^.

The observations obtained during our experiments—such as the sharp pressure rise and the cooling of system components—support this mechanism: the endothermic evaporation absorbs excess heat, preventing thermal degradation of the soft-bodied projectile. This process is associated with vapor explosion mechanisms, including thermal detonation (rapid superheating leading to detonation-like wave propagation) and interfacial fragmentation (hot gases breaking *LN₂* into droplets, thereby increasing the surface-to-volume ratio *S/V ~ 1/r*)^48^.Instabilities such as Rayleigh–Taylor and Landau mechanisms further enhance evaporation by increasing interfacial roughness, as evidenced by evaporation rates observed to be two orders of magnitude higher than those predicted by conventional bubble growth models^49,50^.

To intensify vapor generation, our setup provides: (i) fragmentation of the liquid–gas interface by turbulent thermite combustion gases; (ii) enhanced contact due to the specific design of the apparatus; and (iii) suppression of stable film boiling through pulsed heating from the igniter, which promotes direct interaction between the liquid and hot particles^51,52^. Future Computational Fluid Dynamics (CFD) modeling may further refine these conditions by accounting for Rayleigh–Taylor instabilities during droplet fragmentation^53,54^.

### Experimental methodology and algorithm of actions

**Fig. 3:**
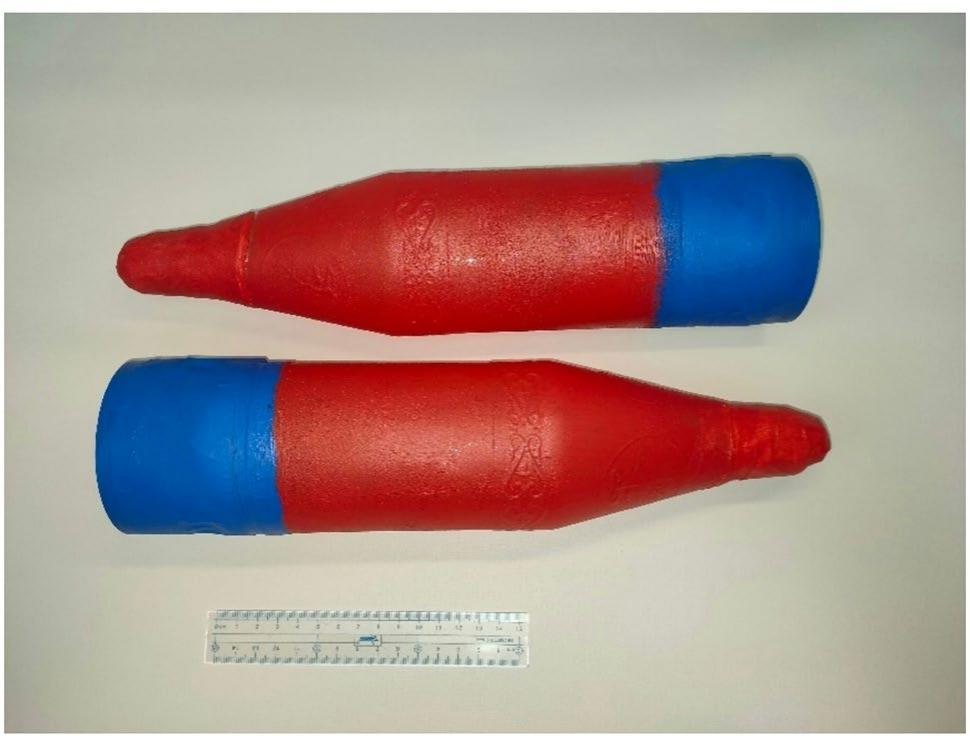
A mock-up of a structurally weak projectile, providing imitation of the mass and geometry of the ammunition during testing (with an applied scale ruler).

The principle and algorithm of the proposed installation include several successive stages. First, the projectile with the obturator is inserted into the barrel and sent to the loading part *b* (see Fig. 1). The projectile itself is a mock-up of a structurally weak projectile made of PET material, which is easily destroyed by strong shock waves. The appearance of the projectile mock-up is shown in Fig. 3. The body has a streamlined hollow shape of a projectile-like configuration. The hollow design allows you to set the required weight by filling the internal volume with bulk material. As noted earlier, the studies used projectiles weighing about 1 kg, which corresponds to the mass of projectiles used in certified pneumatic guns.

**Fig. 4:**
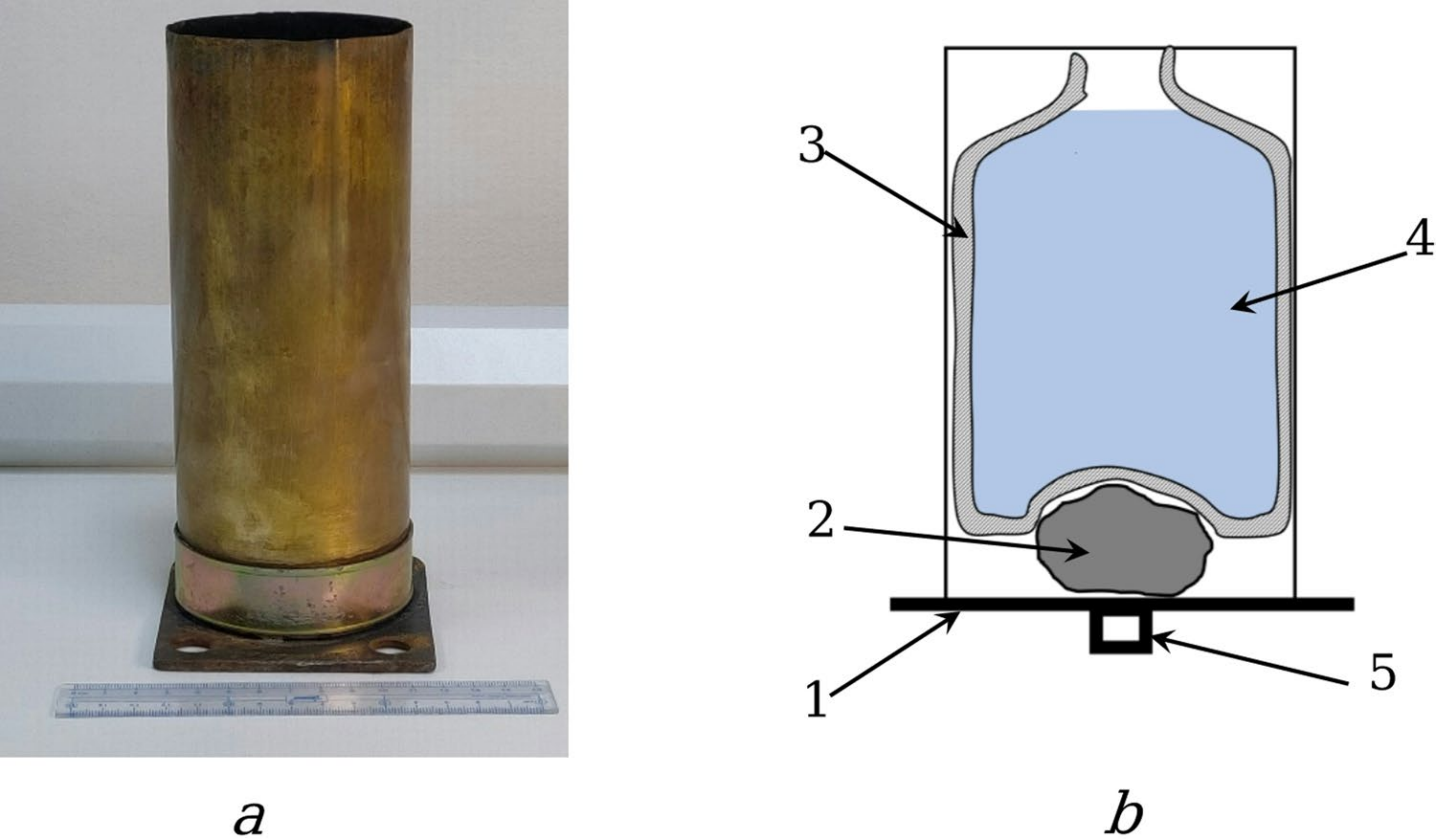
Photo of the cartridge case and diagram of its design with the location of the propellant charge: **a** - general view of the brass cartridge case with a mounting flange (in the foreground - a scale ruler); **b** - cross-sectional diagram (1 - support base; 2 - thermite mixture 2, packed in a paper shell; 3 - thermos cup; 4 - *LN*_*2*_; 5 - primer cap).

Next, the propellant charge, consisting of *LN*_*2*_ and a thermite mixture, is placed in a specially made cartridge case. The cartridge case is a strong metal cylinder made of brass. Figure 4 shows a photo of a real cartridge case (a) and a basic diagram of the arrangement of the propellant charge elements (b) in the cartridge case. At the bottom of the cartridge case 1 is a thermite mixture 2, packed in a paper shell. A thermocup 3 with *LN*_*2*_ 4 is inserted above the thermite mixture. *LN*_*2*_ is poured into a polyethylene bag, which is placed inside the thermocup. The thermocup is made of a building heat-insulating material - foamed polyethylene. It has a narrowed neck, which does not allow *LN*_*2*_ to spill out when the barrel is tilted too much. And the primer-igniter 5 is also located at the bottom of the cartridge case opposite the thermite mixture. This arrangement of the propellant charge elements ensures their effective interaction at the moment of ignition.

The loaded cartridge case is installed in the base of the barrel and held in place by a precise fit and the breech mechanism. Once the breech is closed, the firing mechanism is fully prepared to fire. When the trigger is pressed, the firing pin is triggered, igniting the primer, which in turn ignites the thermite mixture, producing hot gases. These gases directly affect the *LN*_*2*_, breaking it into fine droplets. Rapid heat transfer from the hot gases to the droplets causes them to instantly boil and expand sharply, creating high pressure. This pressure pushes the projectile down the barrel and gives it the required initial velocity.

After firing, the cartridge case remains in the barrel and is then removed for reloading. In addition to housing the propellant charge and the igniting cap, the cartridge case performs the important function of sealing the barrel at the moment of firing, reliably holding the resulting working gases and directing their action exclusively to throwing the projectile forward, preventing leaks through the bolt.

To assess the thermal load on the projectile shell, the temperature of the outer surface of the shell was measured after each shot. In the case of using only the thermite mixture without adding *LN*_*2*_ (for example, 3 g of thermite and 0 ml of nitrogen), the shell noticeably heated up, and its temperature reached about 60 °C. However, when firing with the addition of *LN*_*2*_, the shell temperature, on the contrary, decreased: depending on the volume of cryofluid, it ranged from + 20 °C to − 20 °C. Thus, a pronounced inverse relationship was observed between the volume of *LN*_*2*_ and the final temperature of the working gas. This is due to the fact that during evaporation, *LN*_*2*_ actively absorbs the heat released during combustion of the thermite mixture, and thus limits the heating of the outer shell of the shell. This effect explains why a plastic shell located above the ignition zone is not subject to thermal destruction.

### Measuring the average speed of a body in a barrel

**Fig. 5:**
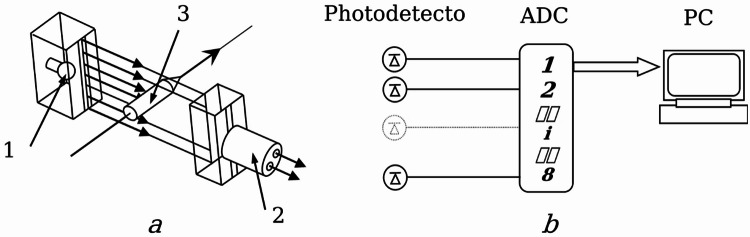
Photoelectric projectile flight detection system:. **a** – optical unit (1 – radiation source; 2 – photodetector module; 3 – optical beams intersected by the projectile); **b** – connection diagram of photodetectors to an analog-to-digital converter (ADC) and a computer (PC).

The motion of a body accelerated by pneumatic pressure is studied by a chronographic method formed from infrared photodiodes. Such a chronograph allows for precise measurement of the projectile velocity and study of its motion characteristics. The chronograph design is quite compact and consists of 8 optical frames or “gates” installed on the projectile’s flight path. Each frame includes a light emitter (infrared diode) and a receiver — a photo sensor — and is located at a specified distance from each other. When a projectile flies through an optical frame, it interrupts the light beam and the sensor records the moment of operation. Figure 5 shows a diagram of the optical frame (a) and a diagram of the organization of the registration system from optical sensors (b). The optical frame (see Fig. 5a) consists of a light source 1, powered by direct current, and a photo recorder 2. Since the LEDs have a narrow radiation angle, and the light from the source is completely blocked by the projectile 3, the light source and receiver are used without a collimator.

Then the signals from the photo recorders are fed to the analog-to-digital converter (ADC), which is shown schematically in Fig. 5b. As the projectile flies through each frame, the ADC records the exact time of the light beam overlap. Knowing the exact distance between the frames and the time difference, it is possible to calculate the average projectile speed at each interval of the path it has traveled. The signals from the sensor at the moments of operation are recorded on a personal computer (PC) using a multi-channel analog-to-digital converter of the E14-140 M type with PowerGraph-3.3 software as telemetry. The results obtained from 8 optical frames are recorded in a PC for further analysis via 8 independent channels using the ADC. The frequency of recording the moments of flight was 10 kHz. Thus, the use of a multi-channel optical chronograph ensures recording the moments of the projectile passing through each section and allows for determining its speed of movement inside the installation with high accuracy.

The calculations took into account the measurement error associated with the discreteness of time recording and the possible deviation of the projectile from the axis of symmetry. The error in determining the flight time did not exceed ± 0.01 ms, which, with a base interval of 500 mm, gave a maximum relative error in measuring the speed of about ± 10%. To increase reliability and assess reproducibility, each experiment was conducted three times under the same initial conditions. The obtained speed values were averaged based on the results of repetitions. A detailed uncertainty budget, including all contributing factors and their quantitative evaluation, is provided in Supplementary Information 1.

**Fig. 6:**
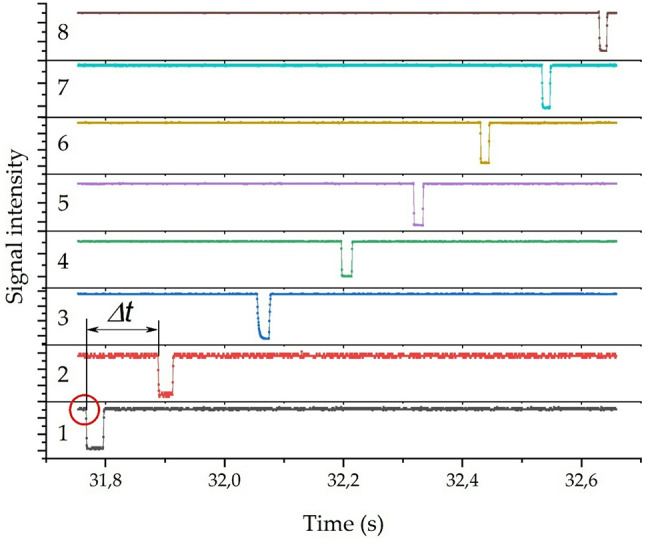
Time dependences of photodetector signals on light intensity for eight successively positioned optical frames along the barrel of the installation. The signal dip indicates the moment the projectile intersects the beam; the time interval *Δt* between successive dips determines the projectile’s velocity.

Figure 6 shows an example of recording signals from eight optical frames via independent channels. The graph shows the dependence of light intensity signals on time for eight optical sensors installed sequentially along the pipe shaft. Each sensor records the signal level - when a projectile crosses the light beam, the beam is briefly blocked, which is recorded as a sharp signal drop on the corresponding curve. For example, the curve for the first sensor (marked as “1”) shows a drop (marked with a circle) - this means that the projectile crossed the first sensor at this very moment. Similarly, for the second sensor (“2”), the next drop records the moment the projectile passes through the second beam. By determining the exact time the signal drop begins for the first and second sensors, it is possible to calculate the time interval during which the projectile moved from the first sensor to the second. Knowing this time and the distance between the sensors, the average projectile velocity in the section between them is calculated using the formula:

1 $$\:\:\begin{array}{*{20}{c}} {v = \frac{L}{{\Delta \:t}}} \end{array}$$

where is the distance between the sensors, and Δ is the time interval between overlapping moments.

The choice of this type of optical chronograph is due to the fact that it has high accuracy, no mechanical contact with the flying body, does not affect the movement of bodies and the frames are not destroyed after the body passes, which significantly facilitates and speeds up the experiments.

## Energy balance assessment

In this section, to test the feasibility and internal consistency of the concept, we conducted a compact energy analysis. The evaluation quantitatively reflects the distribution of thermite’s thermal energy on the evaporation of *LN*_*2*_.

We partition the thermite chemical energy as:

1 $$\:{Q}_{th}=\:{Q}_{evap}+{Q}_{heat}+{W}_{proj}+{Q}_{loss}$$

where.

$\:{Q}_{th}$ - thermite heat release (chemical enthalpy);

$\:{Q}_{evap}$ - latent heat to vaporize *LN₂*.

$\:{Q}_{heat}$ - sensible heating of produced N₂ above boiling.

$\:{W}_{proj}$ - mechanical work on projectile;

$\:{Q}_{loss}$ - losses to chamber walls, radiation, unvaporized liquid, and kinetic energy of expelled gas.

In the zero-order approximation, the evaporation of *LN₂* induced by the thermite reaction occurs significantly faster than the projectile motion, so the initial pressure rise can be approximated as nearly isochoric over a short time interval.

The expansion process is assumed to be adiabatic, i.e., *PV*^*γ*^ *= const*. The pressure behind the projectile is considered spatially uniform, since the speed of sound in the hot gas is much higher than the projectile velocity. Leakage of the working fluid is assumed to be negligible, and the frictional force is accounted for as a subtractive term *F*_*fric*_ in the equation of motion. The simplified phase-dynamics model considers the liquid-to-vapor transition without a detailed description of microscopic mechanisms such as nucleation, bubble growth, fragmentation, or supercooling. It is assumed that the phase transition completes instantaneously compared to the characteristic timescale of projectile motion, and that the gaseous working fluid can be treated as thermodynamically equilibrated and spatially uniform in temperature and pressure within the chamber volume. This approximation enables an analytical description of the launcher’s internal ballistics and allows estimation of the key energetic relationships required for interpreting the experimental data.

Then the motion of the projectile is described by the following equations of motion:

2 $$\:{m}_{p}\ddot{x}=\left(P\left(t\right)+{P}_{atm}\right)A-{F}_{Fric}$$

where *A* is the cross-sectional area of the trunk, $\:{m}_{p}$ is the mass of the projectile.

Taking into account the following useful relationship $\:V\left(x\right)={V}_{0}-Ax$ nd the mean pressure $\:\stackrel{-}{P}$, the work equation can be written as follows:

3 $$\:{W}_{proj\:}\approx\:\:\stackrel{-}{P}AL;\:\frac{1}{2}{m}_{p}{v}^{2}\:\approx\:\:{W}_{proj\:}\:$$

Hence, a convenient estimate for the required mean pressure can be expressed as:

4 $$\:\stackrel{-}{P}\approx\:\:\frac{{m}_{p}{v}^{2}}{2AL}\:\:$$

The corresponding velocities for *m*_*p*_:

5 $$\:v\approx\:\:\sqrt{\frac{2{W}_{proj}}{{m}_{p}}}\:$$

To verify the energy consistency and physical feasibility of the system, an order-of-magnitude estimate was performed using representative experimental parameters. We assume a thermite mass of ≈ *100 g* and an *LN₂* mass of ≈ 0.4 *kg* (500 *ml*). With a thermite specific energy of ≈ *3.9 kJ·g*^− 1^, the total chemical energy available is ≈ 396 *kJ*. *80 kJ* of this energy is required to vaporize the *LN₂* (at specific heat of vaporization ≈ *200 kJ ·kg*^− 1^), and the remaining energy is used to heat the gas, expand it and perform mechanical work on the projectile.

These estimates rest on the zero-order assumptions of near-isochoric initial heating (thermite-driven, fast *LN₂* evaporation), adiabatic gas expansion during the run, negligible leakage and modest friction. Under these assumptions the predicted muzzle speed lies in the *~ 290–460 m·s*^− 1^ interval. In practice, therefore, the *290–460 m·s*^− 1^ range should be interpreted as an order-of-magnitude design target rather than a precise prediction: (a) lower velocities are expected if phase-change kinetics or leakage reduce the effective pressure, (b) higher velocities require either higher mean pressure (more thermite or better confinement) or a longer barrel to extract more work. Finally, these results show that the *thermite–LN₂* concept can generate practical projectile speeds while offering a tunable trade-off between peak pressure and pulse duration through the thermite: *LN₂* matching, which is critical for achieving gentle acceleration of fragile payloads.

## Results and discussion

**Fig. 7:**
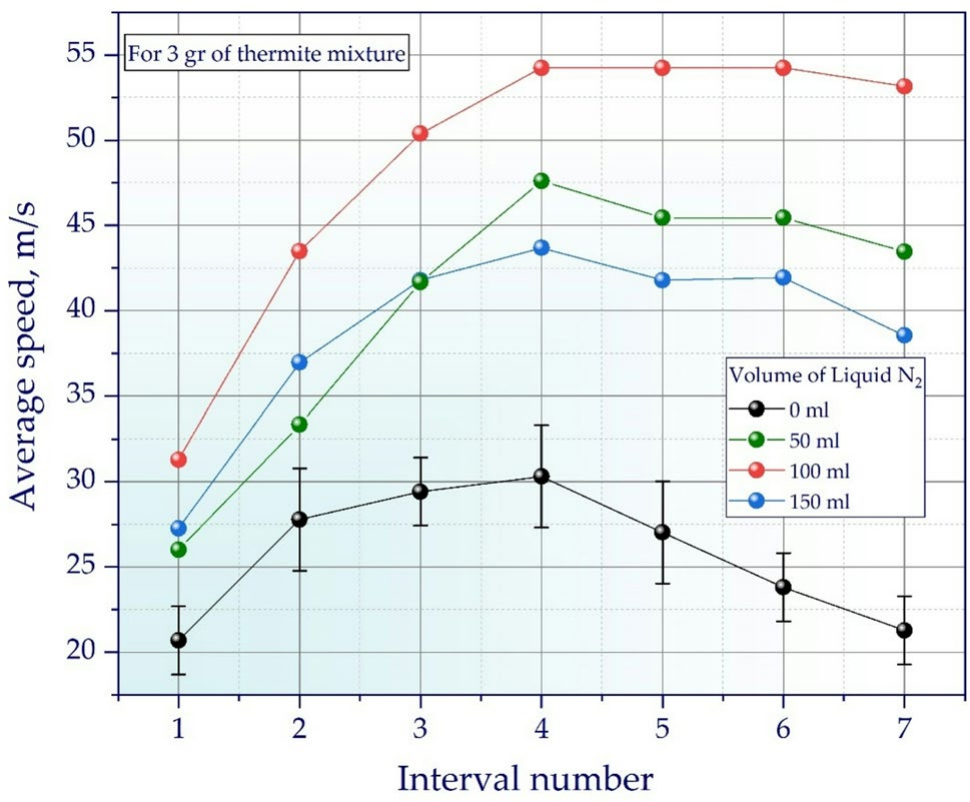
Graph of the average projectile velocity at fixed intervals along the barrel for 3 g of thermite mixture and various volumes of *LN*_*2*_ (0, 50, 100, and 150 ml). The dots represent experimental data, and the vertical bars represent confidence intervals.

Figure 7 shows the dependence of the obtained average velocity on the interval for 3 g of thermite mixture, but with different amounts of *LN*_*2*_. The volume of *LN*_*2*_ was varied from 0 ml to 150 ml in increments of 50 ml. The graph shows that with a fixed amount of thermite mixture (3 g), changing the volume of *LN*_*2*_ significantly affects the dynamics of acceleration of the working fluid inside the barrel. The experiment conducted only with thermite mixture without *LN*_*2*_ is basic. Without adding *LN*_*2*_, the speed remains at a minimum level: the initial growth is replaced by a decline after the fourth interval, which indicates a rapid decrease in pressure and low acceleration efficiency. When adding 50 ml of *LN*_*2*_, the average speed increases more intensively and reaches a maximum by the fourth interval, then begins to gradually decrease, which indicates a longer maintenance of pressure in the barrel. The highest speeds are achieved with a *LN*_*2*_ volume of 100 ml: here, a sharp increase is observed up to the fourth interval with subsequent stabilization at a high level, which indicates an optimal ratio of the amount of working fluid and thermal energy from the thermite mixture. With an increase in volume to 150 ml, the speed also increases, but is slightly inferior to the maximum value at 100 ml. While an earlier and gradual decrease in speed is visible at the last intervals, which may be due to a lack of thermal energy of 3 g thermite for complete evaporation of 150 ml of nitrogen and maintaining optimal pressure throughout the entire flight time of the projectile in the barrel.

Thus, it is experimentally confirmed that the selection of the amount of *LN*_*2*_ allows controlling the internal ballistics of the installation, achieving maximum speeds and improving the overall performance of the system.

**Fig. 8:**
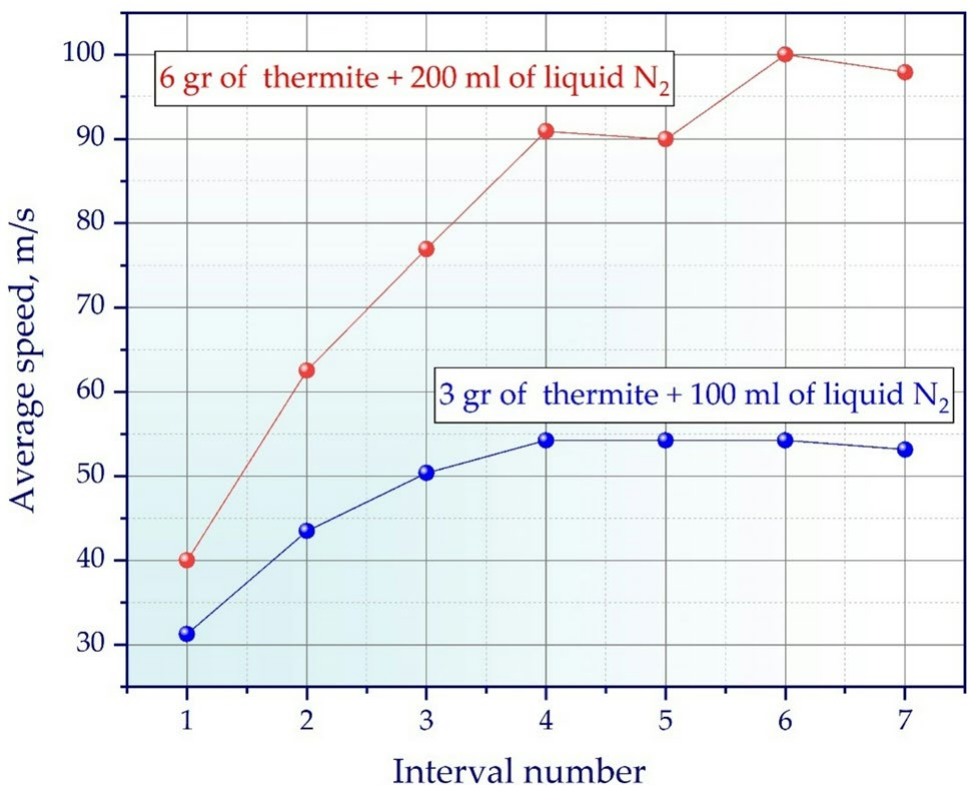
Comparative graph of the change in the average velocity of a projectile along the barrel for two modes: 3 g of thermite mixture + 100 ml of *LN*_*2*_ and 6 g of thermite mixture + 200 ml of *LN*_*2*_.

Figure 8 shows a comparison of the acceleration of the working fluid inside the barrel for two different combinations of thermite mixture mass and *LN*_*2*_ volume. One curve (blue line with round markers) illustrates the previously determined optimum mode using 3 g of thermite mixture and 100 ml of *LN*_*2*_. The second curve (red line with round markers) shows how the acceleration pattern changes with a two-fold increase in thermite charge mass — to 6 g — and with a corresponding increase in the *LN*_*2*_ volume to 200 ml. This curve corresponds to the maximum velocity value for 6 g of thermite mixture from a change in the nitrogen amount from 0 to 350 ml with the same step as for 3 g of thermite — 50 ml. We do not provide these data here for brevity, since the data for 3 g are obvious on the graph of Fig. 7 (A graph of the average projectile velocity at fixed time intervals along the barrel for 6 g of thermite mixture and different volumes of *LN*_*2*_ is given in Supplementary Information 2).

Thus, from the graph of Fig. 8 it is clear that with a doubled charge the speed increases significantly faster and reaches higher values at all intervals of the barrel. With 3 g of thermite, the velocity increases and reaches a plateau by the fourth interval, stabilizing around 55 m/s until the projectile exits the barrel. In contrast, with a larger charge (6 g), acceleration continues up to the sixth interval, and the speed peaks at over 90 m/s, decreasing only slightly near the end.

The nature of the curves shows that a larger volume of *LN*_*2*_ combined with an increased mass of the thermite mixture ensures a longer maintenance of high pressure in the barrel and a more uniform transfer of energy to the working fluid. As a result, the effective acceleration time is extended, and the maximum speed increases significantly. This confirms that increasing the charge without changing the proportions between the solid and liquid components makes it possible to significantly improve the ballistic characteristics of the installation, while maintaining acceleration stability along the entire length of the barrel.

The experimental data we obtained give grounds to assume that the revealed consistencies can be extended to other combinations of the thermite charge mass and the amount of *LN*_*2*_. Since the principle of interaction of the thermite thermal energy and the cryogenic charge effect is preserved, the results can be extrapolated to a wider range of parameters. Based on this, it is possible to construct a qualitative (schematic) graph, which can illustrate the predicted change in the average projectile velocity for different variants of pressure distribution inside the barrel. This approach not only helps explain the obtained facts, but also to set guidelines for choosing the optimal operating modes of the installation when changing the composition and characteristics of the propellant charge.

**Fig. 9:**
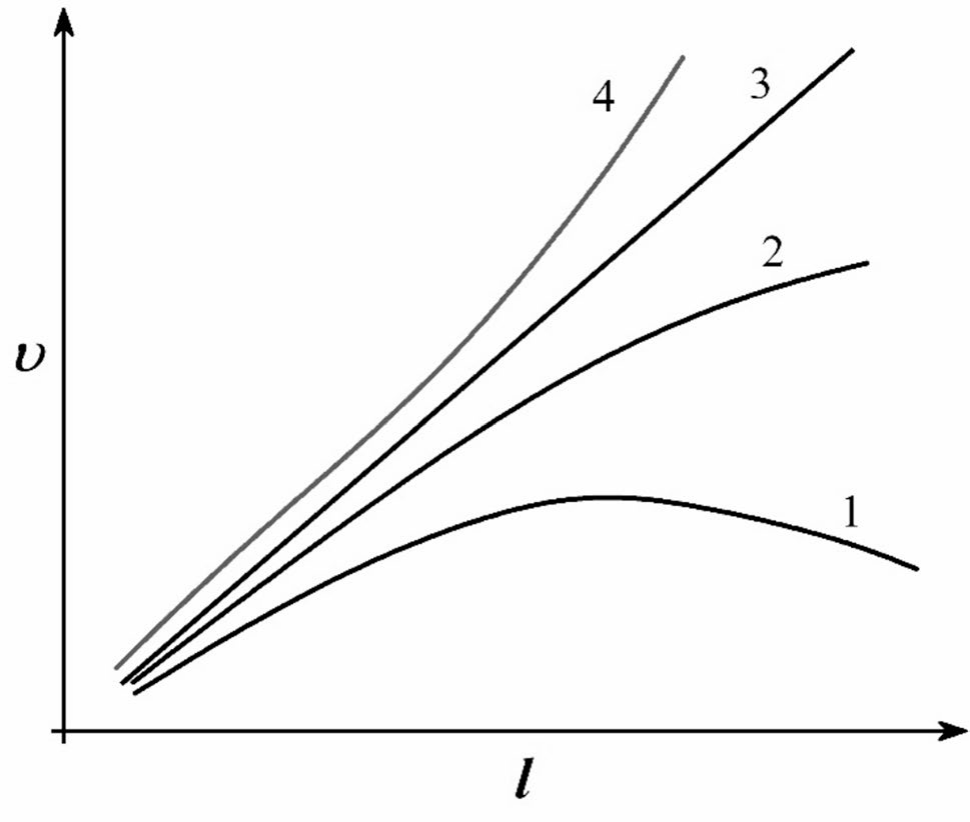
Schematic graph of the change in the average velocity of the projectile () along the barrel for different variants of pressure distribution inside the channel: 1 - rapid pressure drop with loss of the accelerating effect; 2 - moderate increasing pressure distribution; 3 - almost linear increase in velocity at stable pressure; 4 - intensive increase in velocity at increased pressure.

This idea is shown in Fig. 9 using schematic graphs of the change in the average velocity of the projectile along the barrel for different variations of the propellant charge elements. These curves can be used to show the effect of pressure distribution on the projectile at different moments of its flight. For example, the first curve represents a case of uneven pressure distribution. The projectile experiences a strong initial impulse, and its velocity increases rapidly. However, the pressure drops before the projectile exits the barrel, and some of the energy is lost - as a result, the velocity reaches its maximum and begins to decrease even before exiting. And the second curve shows a more favorable regime, when the pressure is maintained longer and is distributed more evenly along the movement - due to this, acceleration continues almost to the end of the barrel, and the velocity increases more stably. The third curve corresponds to an almost ideal case: the pressure acts uniformly throughout the entire movement of the projectile, due to which the velocity continuously increases and reaches its maximum value at the exit. The fourth curve demonstrates a special case, when the peak pressure occurs closer to the end of the barrel channel, providing a sharp increase in velocity immediately before departure.

Thus, the experimental fact obtained by us, shown in Fig. 8, corresponds to the dynamics of the body motion, which is shown in Fig. 9 by curves 1 and 2. These dependencies show how important it is to correctly form the pressure along the barrel length for the full use of the charge energy and achieving the required ballistic characteristics.

The most optimal and required ballistic characteristics for the installation we have developed is, of course, uniform maintenance of pressure in the barrel until the projectile flies out, that is, the correspondence of the experimental results with curve 3 of the qualitative graph of Fig. 9. And in the future, different angles of this curve will correspond to different speeds. An increase in the angle leads to an increase in the average speed along the barrel length. The study of the designated problems will be published in the following publications.

## Comparative analysis with existing methods

For an objective assessment of the position of our pneumatic launcher among existing avalanche-control methods and technologies, a comparative analysis with these methods is required, even if their actuation principles differ. Such a comparison permits an approximate evaluation of the strengths and limitations of the proposed approach. Table 1 below lists the parameters most relevant for practical applications: pressure-generation principle, working medium, nature of peak loads, effect on the projectile casing, mobility, and environmental safety.

**Table 1:** Comparative characteristics of remote avalanche-initiation methods.

Parameter	Traditional artillery [7,14,17]	Gas Avalauncher (pneumatic) [12,19,20]	Solid-propellant/powder systems [8,13]	Proposed cryo-pneumatic launcher
**The principle of pressure creation**	Detonation/combustion of charges	Quick use of receiver (compressed gas)	Solid fuel combustion/gas generation	Explosive phase transition of *LN₂* initiated by thermite
**Working fluid**	Gaseous combustion products	Compressed air/gas from the receiver	Hot gases	Nitrogen vapor/gaseous product produced by explosive boiling of *LN₂*
**Peak pressure type**	High impulse (shock) loads	Controlled (depending on valve)	High, closer to impulse	Adjustable pressure pulsations, without strong shock peaks
**Effect on the soft shell of the projectile**	High probability of destruction	Average (with careful adjustment)	High	Low (soft, even acceleration with the correct *LN₂*/thermite ratio)
**System mobility**	Low (heavy weapons)	Average (receiver + cylinders)	Average	High (no heavy receiver)
**Environmental safety**	Low (fragments, explosive residues)	Average	Low/Average	High (no traditional explosives, minimal metal fragments)

The comparison shows that the key advantage of the proposed system is its combination of mobility, adjustable pressure profile, and smooth acceleration for structurally fragile projectiles. Unlike artillery or solid-propellant systems, where high impact loads and fragmentation remain challenges, our method provides more controlled energy transfer through the endothermic evaporation of *LN₂*. Compared to gas-powered Avalaunchers, there is no need for a heavy receiver or high-pressure cylinders, improving portability and logistics. Numerical values for peak pressures, velocities, and ranges in the literature vary widely depending on the model and test conditions; for this reason, the table uses qualitative comparisons and cites sources for each category. Accurate quantitative comparisons require standardized field testing with consistent protocols and identical measurement methods. This issue will be the subject of our future work.

## Conclusion

This paper presents the results of the development, creation and experimental study of a functioning laboratory prototype of a pneumatic throwing installation using *LN*_*2*_ as a working fluid. The developed laboratory prototype made it possible to experimentally test the principle of using an explosive phase transition of *LN*_*2*_, which ensures the rapid formation of high pressure in the barrel.

During a series of experiments, it was found that with a thermite mixture mass of 3 g and the addition of 100 ml of *LN*_*2*_, the optimal ballistic characteristics for this amount of thermite are achieved. The average projectile velocity in the barrel increases to 55 m/s and remains at this level almost until the projectile exits the barrel. With an increase in the mass of the thermite mixture to 6 g and the volume of nitrogen to 200 ml, the speed increases more intensively and reaches a peak of over 90 m/s, which demonstrates the possibility of controlling internal ballistics by changing the parameters of the propellant charge.

The results confirm that the proposed method of creating pressure in the developed installation allows avoiding destruction of a soft-bodied projectile during firing, reducing peak loads and at the same time ensuring sufficient range of delivery to the desired point of explosives for initiating controlled snow avalanches. In comparison with traditional artillery systems, the proposed installation is compact and lighter, which ensures its mobility.

The results of this research serve as a basis for further improvement of the design and optimization of the operating mode of the installation. This includes the study of options for full compliance of the pressure profile in the barrel with the ideal ballistic characteristic, which will allow the most efficient use of the charge energy and expand the scope of application of the installation, i.e. use it not only for avalanche protection, but also for launching small-sized unmanned reconnaissance aircraft.

## Supplementary Information

Below is the link to the electronic supplementary material.

Supplementary Material 1

## Data Availability

The datasets used and/or analyzed during the current study are available from the corresponding author on reasonable request.
