# Supplementary material for: Experimental study of a cryogenic pneumatic thrower for launching soft projectiles in avalanche protection
Source: Sci Rep. 2025 Nov 27;15:42432. doi: 10.1038/s41598-025-26523-x (PMC12660976; doi:10.1038/s41598-025-26523-x)
Supplement: Supplementary file 1 — Supplementary Material 1 [file 41598_2025_26523_MOESM1_ESM.zip › Supplementary Information 1.docx]

**Supplementary Information 1.**

**Statistical Analysis and Uncertainty Evaluation**

The calculations accounted for the main sources of measurement uncertainty associated with (i) the discretization of time recording and (ii) possible deviation of the projectile trajectory from the optical axis between the infrared (IR) sensors. The error in determining the flight time did not exceed ±0.01 ms, which, with a base interval of 500 mm, corresponds to a maximum relative velocity error of about ±10%.

To increase reliability and assess reproducibility, each experiment was conducted three times under identical initial conditions. The obtained velocity values were averaged, and the standard deviation (SD) and standard error (SE) were calculated. Confidence intervals at the 95% probability level were determined using the Student’s t-factor for n - 1 = 2 degrees of freedom.

An uncertainty budget was compiled for the time–velocity measurement chain, including contributions from baseline calibration, timer resolution, electronic jitter, signal threshold noise, and projectile misalignment. The combined standard uncertainty was evaluated using the root-sum-square (RSS) method, and the expanded uncertainty was determined with a coverage factor of *k = 2*.

| **Source of uncertainty** | **Type** | **Estimate (1σ)** | **Relative contribution, %** |
| --- | --- | --- | --- |
| Time measurement discretization (Δt) | B | ±0.01 ms | 8.0 |
| IR gate spacing calibration (ΔL) | B | ±0.5 mm | 1.0 |
| Projectile trajectory deviation | A | ±2 mm | 2.0 |
| Electronic jitter and threshold noise | A | ±0.005 ms | 2.5 |
| Environmental factors (temperature, humidity) | B | ±0.5 % | 0.5 |
| Combined standard uncertainty (RSS) | — | — | 2.0 |
| Expanded uncertainty (k = 2) | — | — | ≈ 3.5 % |

The resulting expanded uncertainty of ±3.5% corresponds to an absolute uncertainty of approximately ±12–15 m·s⁻¹ in the measured velocity range (300–450 m·s⁻¹). The repeated trials demonstrated good reproducibility: the relative standard deviation did not exceed 5%, confirming the stability of both the optical measurement system and the overall internal-ballistic process.
