# Supplementary material for: Experimental study of a cryogenic pneumatic thrower for launching soft projectiles in avalanche protection
Source: Sci Rep. 2025 Nov 27;15:42432. doi: 10.1038/s41598-025-26523-x (PMC12660976; doi:10.1038/s41598-025-26523-x)
Supplement: Supplementary file 1 — Supplementary Material 1 [file 41598_2025_26523_MOESM1_ESM.zip › Supplementary Information 2.docx]

**Supplementary Information 3.**


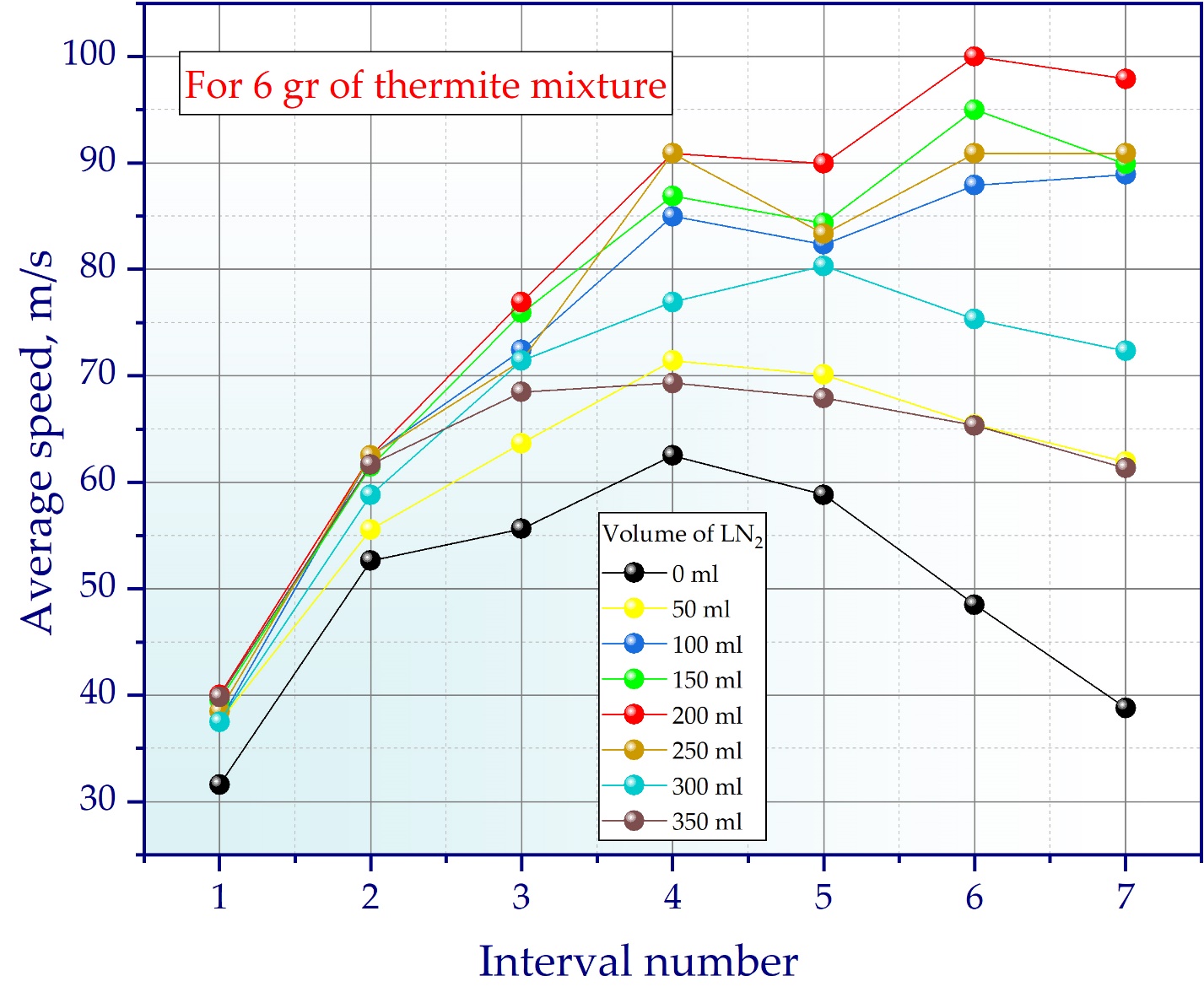


Fig. S1 – Graph of the average projectile velocity at fixed intervals along the barrel for 6 g of thermite mixture and various volumes of liquid nitrogen (0, 50, 100, 150, 200, 250, 300 and 350 ml)
